# Recessive *VARS2* mutation underlies a novel syndrome with epilepsy, mental retardation, short stature, growth hormone deficiency, and hypogonadism

**DOI:** 10.1186/s40246-017-0124-4

**Published:** 2017-11-14

**Authors:** Abdulaziz Alsemari, Banan Al-Younes, Ewa Goljan, Dyala Jaroudi, Faisal BinHumaid, Brian F. Meyer, Stefan T. Arold, Dorota Monies

**Affiliations:** 1 Department of Neurosciences, King Faisal Specialist Hospital and Research Centre, Riyadh, Saudi Arabia; 2 Department of Genetics, King Faisal Specialist Hospital and Research Centre, MBC 03, PO Box 3354, Riyadh, 11211 Saudi Arabia; 3 Saudi Human Genome Project, King Abdulaziz City for Science and Technology, Riyadh, Saudi Arabia; 4 King Abdullah University of Science and Technology (KAUST), Computational Bioscience Research Center (CBRC), Division of Biological and Environmental Sciences and Engineering (BESE), Thuwal, 23955-6900 Saudi Arabia

**Keywords:** Syndromic, Angelman, Mitochondrial, Dysmorphism, Hypogonadism, Vitamin D deficiency

## Abstract

**Background:**

Most mitochondrial and cytoplasmic aminoacyl-tRNA synthetases (aaRSs) are encoded by nuclear genes. Syndromic disorders resulting from mutation of aaRSs genes display significant phenotypic heterogeneity. We expand aaRSs-related phenotypes through characterization of the clinical and molecular basis of a novel autosomal-recessive syndrome manifesting severe mental retardation, ataxia, speech impairment, epilepsy, short stature, microcephaly, hypogonadism, and growth hormone deficiency.

**Results:**

A G>A variant in exon 29 of *VARS2* (c.3650G>A) (NM_006295) was identified in the index case. This homozygous variant was confirmed by Sanger sequencing and segregated with disease in the family studied. The c.3650G>A change results in alteration of arginine to histidine at residue 1217 (R1217H) of the mature protein and is predicted to be pathogenic.

**Conclusions:**

These findings contribute to a growing list of aaRSs disorders, broadens the spectrum of phenotypes attributable to *VARS2* mutations, and provides new insight into genotype-phenotype correlations among the mitochondrial synthetase genes.

## Background

Mental retardation (MR) affects approximately 1–3% of the human population. Both genetic and environmental factors are responsible for causing MR, but in almost 60% of the cases, the actual cause cannot be identified precisely [1, 2]. In developed countries, most severe forms of intellectual disability are thought to have a genetic cause [3]. Mutations in more than 400 genes have been linked to intellectual disability, but most of these mutations have a very low prevalence and their phenotypes are often indistinguishable [4].

A number of single-gene disorders result in mental retardation. Many of these are associated with atypical or dysmorphic physical characteristics. Such conditions include fragile X syndrome, neurofibromatosis, tuberous sclerosis, Noonan’s syndrome, Cornelia de Lange’s syndrome, and Angelman syndrome(AS) [5]. AS is usually considered as a diagnosis in individuals with severe developmental delay, in combination with seizures, ataxia, hypermotoric behaviors, and absence of speech. Ninety percent of these individuals have an identifiable molecular defect that results in the loss of expression of maternally inherited *UBE3A* (encoding E6AP) in the brain [6]. Over the last few years, many novel syndromes that mimic AS have been delineated and it is now recognized that some individuals who were clinically diagnosed with “test-negative AS” have one of these AS-like syndromes instead. Their phenotypes include severe intellectual disability, motor delay, variable degrees of speech delay ranging from being completely non-verbal to being able to speak in short sentences, autistic and maladaptive behaviors, short attention span, and seizures [7].

We report a novel autosomal recessive syndromic disorder with clinical resemblance to AS. The clinical spectrum includes severe mental retardation, ataxia, speech impairment, epilepsy, short stature, microcephaly, flap occiput, protruding tongue, prognathia, tremulous movement of limbs, apparent happy demeanor, easily excitable personality, and excessive chewing mouth behaviors. In addition, there were severe growth hormone deficiency, hypogonadism, and severe osteomalacia. Whole exome sequencing of the affected patients identified a homozygous mutation in *VARS2*.

## Methods

### Subjects and nucleic acid extraction

Four patients from a consanguineous extended family from the same region of Saudi Arabia were examined by a neurologist (AAS), in the department of Neurosciences, King Faisal Specialist Hospital and Research Center (KFSHRC). They were diagnosed with an Angelman-like syndrome. All subjects were enrolled under a KFSHRC IRB-approved (RAC# 13-MED2056-20) protocol with full-written informed consent. Genomic DNA was extracted from peripheral blood samples using standard procedures (Flexi Gene DNA Handbook, Qiagen). Samples were quantitated spectrophotometrically and stored at − 20 °C.

### Linkage and homozygosity mapping

All participating individuals (affected and unaffected) were genotyped using an Affymetrix Axiom array (Affymetrix, Santa Clara, CA, USA) following the manufacturer’s protocol (http://www.affymetrix.com/support/technical/manuals.affx). Resulting genotypes were analyzed for shared runs of homozygosity (ROH) using autoSNPa (http://dna.leeds.ac.uk/autosnpa/). Linkage analysis was performed using the Allegro module of Easy Linkage assuming autosomal recessive inheritance and 100% penetrance (http://genetik.charite.de/hoffmann/easyLINKAGE/index.html).

### Whole exome sequencing and analysis

DNA was amplified to obtain a whole exome Ion Proton AmpliSeq library which was further used for emulsion PCR on an Ion OneTouch System followed by an enrichment process using an Ion OneTouch ES, both procedures following the manufacturer’s instructions (Life Technologies, Carlsbad, CA, USA). The template-positive Ion PI Ion Sphere particles were processed for sequencing on the Ion Proton sequencer (Life Technologies, Carlsbad, CA, USA). Approximately 15–17 Gb of sequence was generated per sequencing run. Reads were mapped to UCSC hg19 (http://genome.ucsc.edu/) and variants identified using the Ion Torrent pipeline (Life Technologies, Carlsbad, CA, USA). The resultant variant caller file (vcf) was filtered to include only homozygous variants within the pre-determined ROH (shared by affecteds only) that were absent from dbSNP, 1000 genomes, approximately 2000 Saudi exomes. Included variants were further selected based upon pathogenicity predicted by Polyphen2 (http://genetics.bwh.harvard.edu/pph2/) and SIFT (http://sift.jcvi.org/). Potential causative variants were confirmed by Sanger sequencing and screened further for familial segregation based upon autosomal recessive inheritance. For confirmation, an amplicon incorporating the candidate variant was amplified and sequenced using a BigDye Terminator kit (Applied Biosystems, Foster City, CA) and run on an ABI 3730xl automated sequencer (Applied Biosystems, Foster City, CA). SeqScape v.2.6 software (Applied Biosystems, Foster City, CA) was used to align sequence data and identify variants.

### Computational structural analysis of mutants

Sequences were retrieved from the Uniprot database. BLAST and SwissModel [8] were used to search for suitable structural templates in the Protein Data Bank (PDB). SwissModel, RaptorX [9], and I-Tasser [10] were used to produce homology models. Models were manually inspected, and mutations evaluated, using the Pymol program (pymol.org). Disorder and secondary structure elements were predicted using RaptorX. Transmembrane helices were predicted using Phobius [11]. Functional information was compiled from various resources, including Uniprot [12], InterPro [13], and publications associated with the model templates used.

### Results

#### Clinical profile

**Fig. 1:**
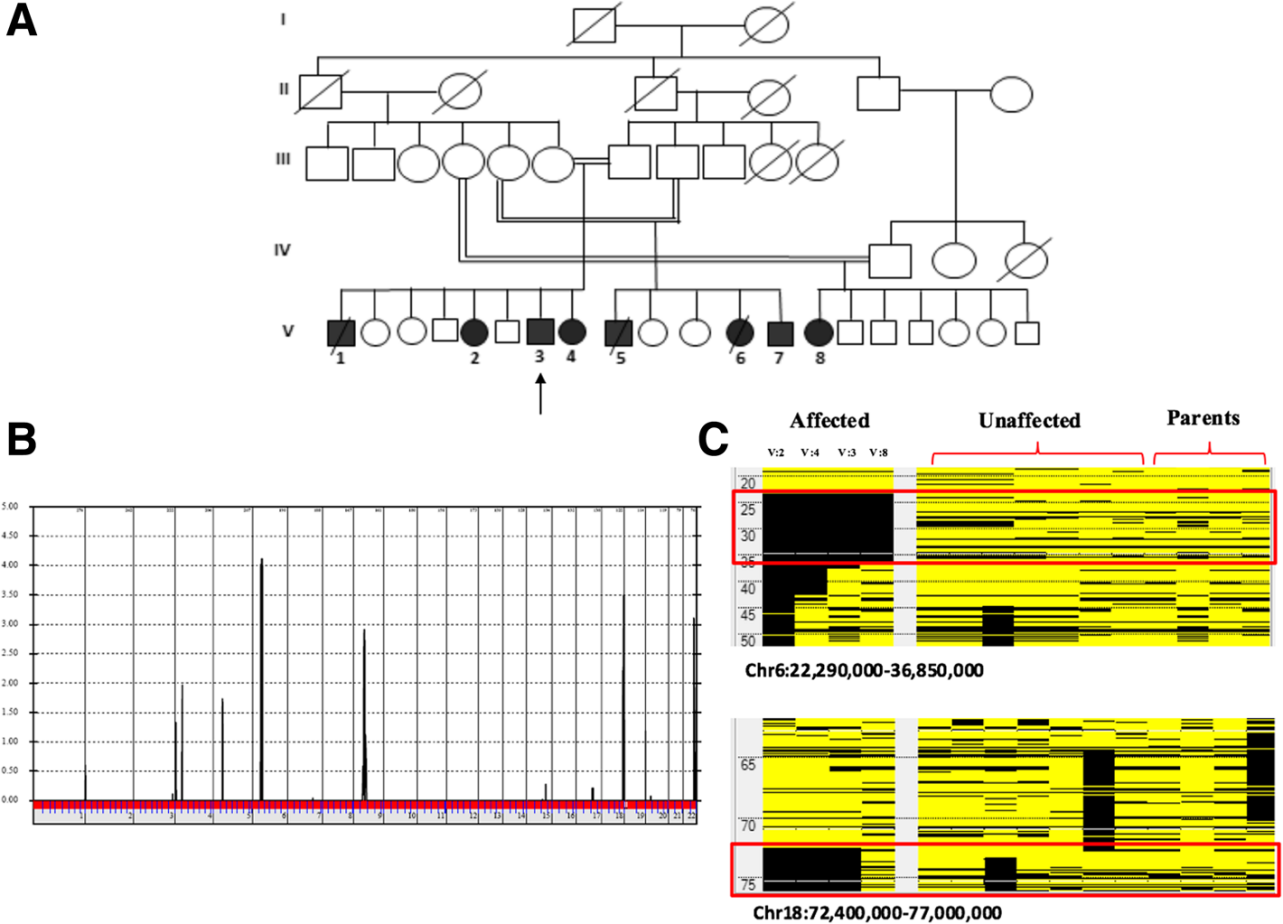
Identification of disease locus on chromosome 6: **a** Pedigree of extended family with a novel syndromic disorder. **b** Genome-wide linkage analysis revealed a peak with a maximum LOD score of 4.11 on chromosome 6. **c** AutoSNPa output for chromosome 6 reveals an ROH (boxed in red) shared among affected members (V:2, V:3, V:4, and V:8) and not present in unaffected individuals or parents. Output for chromosome 18 excludes this locus because the ROH is not shared with affected individual V:10

The index case (V:3) (Fig. 1a), a 23-year-old male was a product of full-term pregnancy and normal delivery. He was well until the age of 4 months when he started to have generalized tonic clonic seizures. At the age of 1 year, the family noticed that the patient had delayed development. He had delayed sitting, delayed standing, and only started to crawl at the age of 2 years. The patient eventually exhibited severe developmental delay and severe speech impairment. The speech impairment was more receptive than expressive. Upon reviewing his family history, there were three other similarly affected siblings (one brother and two sisters). He also has four similarly affected cousins (two males and two females) (Fig. 1a).

**Fig. 2:**
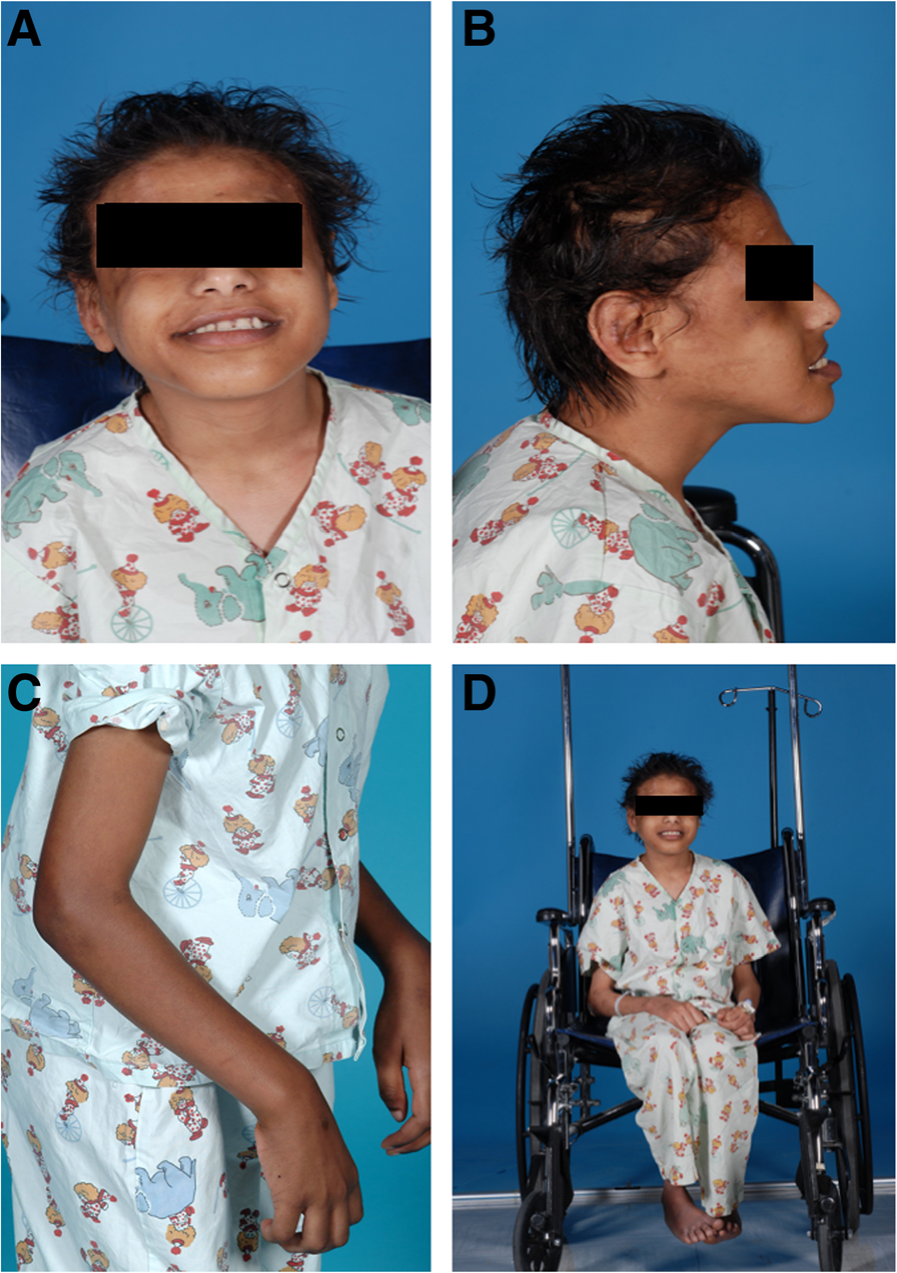
Clinical features: **a** Apparent happy demeanor with easily excitable personality. **b** Microcephaly, prognathia, and wide spaced teeth. **c** There are usually flexed arm positions particularly during movement. **d** Short stature and increasingly chair bound

Affected individuals usually have incoordination of movement until adolescence at which point they stop walking, consequent to flexed joint deformities of the lower limbs. Their behavioral pattern is best described as frequent episodic smiling and an apparent happy demeanor (Fig. 2a), with an easily excitable personality and habitual hand flapping movement. They also have a history of hyperphagia and usually eat a lot more than their normal siblings. Their examination showed severe mental retardation with expressive and receptive aphasia. Their speech and cognition deteriorates to a degree at which they became unable to produce words or to understand commands. There is no facial, axillae, or pubic hair. The affected male had small testicles. They have flat occiput and constant protruding tongue with tongue thrusting. They have microcephaly, prognathia, and wide-spaced teeth (Fig. 2b). During movement of the upper limbs, there are usually flexed arm positions (Fig. 2c). They are of short stature and increasingly become chair bound (Fig. 2d). They typically show an excessive chewing mouth behavior. Their examination showed brisk reflex of the upper and lower limbs. The older affected son (V:1) expired at the age of 23 because of a chest infection.

Laboratory investigations of the index case showed that he had a normal blood count, normal liver function test, and normal electrolytes and renal function test. His peripheral smear morphology was normal. His calcium was low at 2.3 mmol/l, while his phosphate amylase was normal. He had high alkaline phosphatase at 288 U/L (normal range is 40–135). The patient had normal synacthen test and a low IGF level (26 μg/l) (normal range between 116 and 358). IGF binding protein-3 was 1.8 mg/l, which is extremely low (normal range is 3.4–7.8 mg/l). His FSH was 1.8 IU/L and LH was 0.5 IU/L, both in the lower range. The prolactin level and TSH was normal. Testosterone was extremely low < 0.1 nmol/l (normal range is 9.9–27.8). Free androgen index is low < 0.1% (normal range is 14.8–94.8). The 25-hydroxy vitamin D level is undetectable in blood.

Twenty-four-hour urine collection for oligosaccharides was normal, and mucopolysaccharide was negative in the urine. Chromosomal analysis was 46,XY and attributed as no apparent chromosomal abnormalities. FISH analysis to test *SNRPN* deletion within the 15q11.2 locus for AS was negative. The patient also had a growth hormone stimulation test, and the result showed a flat response with a fasting growth hormone of 0.6 m U/L. After 10 min, it was 0.6 m U/L and after 1 h was 1.3 m U/L. This indicated severe growth hormone failure.

A skeletal survey was also done for the patient, and the result was in keeping with metabolic bone disease with evidence of severe osteomalacia and multiple looser zones and fractures, microcephaly, scoliosis, and kyphosis. Serum homocysteine was normal. MRI of the brain was performed and concluded that there were relative atrophic changes seen involving the cerebellum; otherwise, there was no evidence of white matter disease, cortical matter disease, or cortex abnormality.

A muscle biopsy was studied for the index patient. It was interpreted as having type II fiber atrophy with glycogen excess associated with a mild increase in mitochondria. Similar findings were observed in his brother and sister. There were no deficiencies in mitochondrial electron transport chain enzymes. Citrate synthase activity was increased, suggesting increased mitochondrial proliferation.

### Linkage and homozygosity mapping

Linkage analysis (Fig. 1b) localized the disease to a region on chromosome 6 defined proximally by rs743646 and distally by rs34811366 (chr6:4,536,241-6,670,705) with a maximum LOD score of 4.20. Based upon runs of homozygosity shared by all affecteds and absent in all unaffected individuals (Fig. 1c; red rectangle), we refined the critical interval to a 2.1 Mb region (chr6:4,231,133-6,018,243) containing approximately 60 RefSeq genes (hg19).

### Whole exome sequencing and analysis

**Fig. 3:**
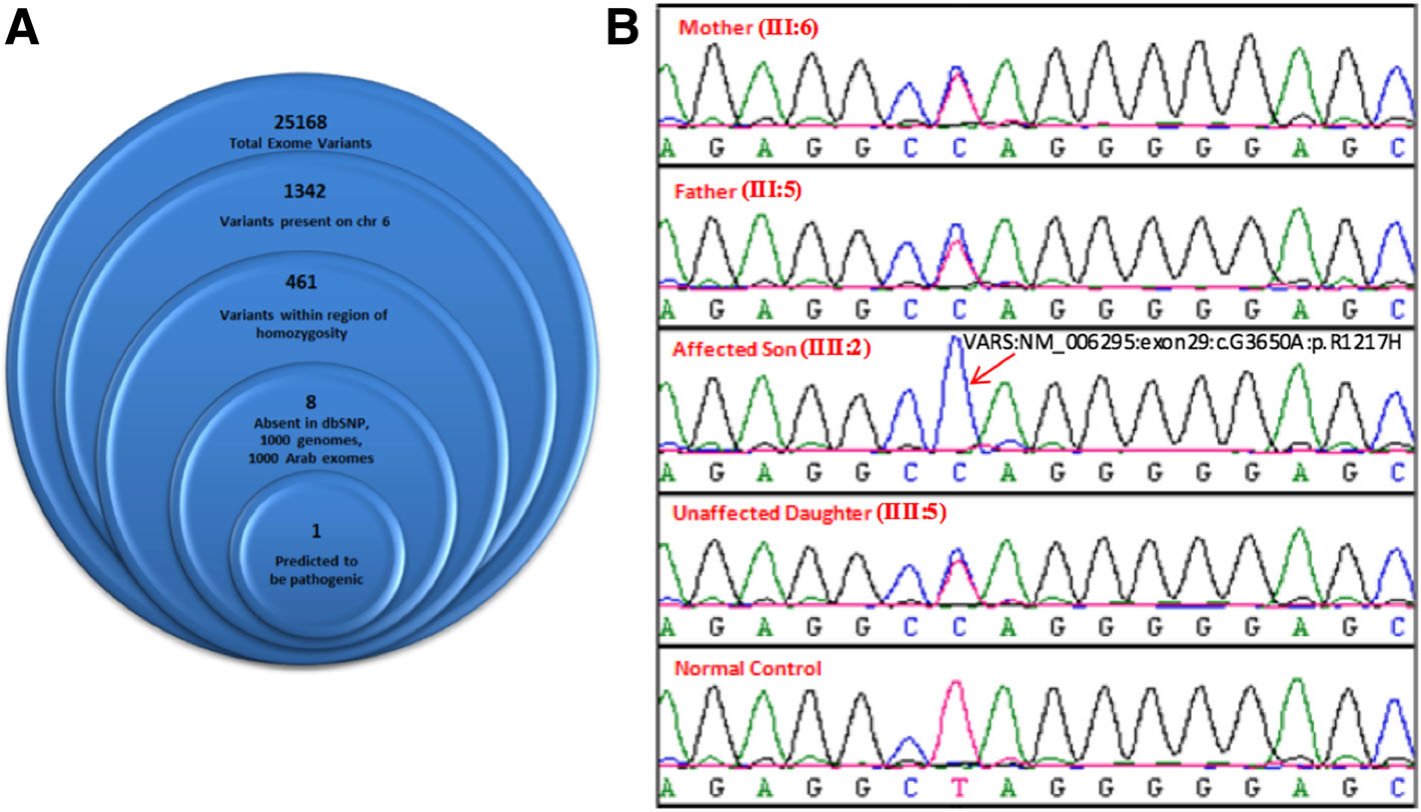
*VARS2* mutation: **a** Filtering strategy used for identification of a causative mutation. **b** DNA electrophoregram with the G>A change in *VARS2* (NM_006295:exon29:c.G3650A:p.R1217H)

Whole exome sequencing of the index case (V:3) identified 25,168 variants relative to hg19, with 1342 variants on chr 6. These were further filtered to exclude all variants present outside our defined ROH (chr 6: 4,231,133-6,018,243) within which we identified 461 variants. By excluding previously reported variants (present in dbSNP, 1000 genomes and 2000 Arab exomes), the list was narrowed to 8. By only focusing on homozygous changes that were non-synonymous, splicing variants, frameshift insertions or deletions, and nonsense variants, we decreased the number of candidate variants to 1. It was a G>A change in exon 29 of *VARS2* (NM_006295:c.3650G>A:R1217H) (Fig. 3a). This homozygous variant was confirmed by Sanger sequencing, and the mutation segregated recessively with disease in the families studied (Fig. 3b).

### Structure and function

**Fig. 4:**
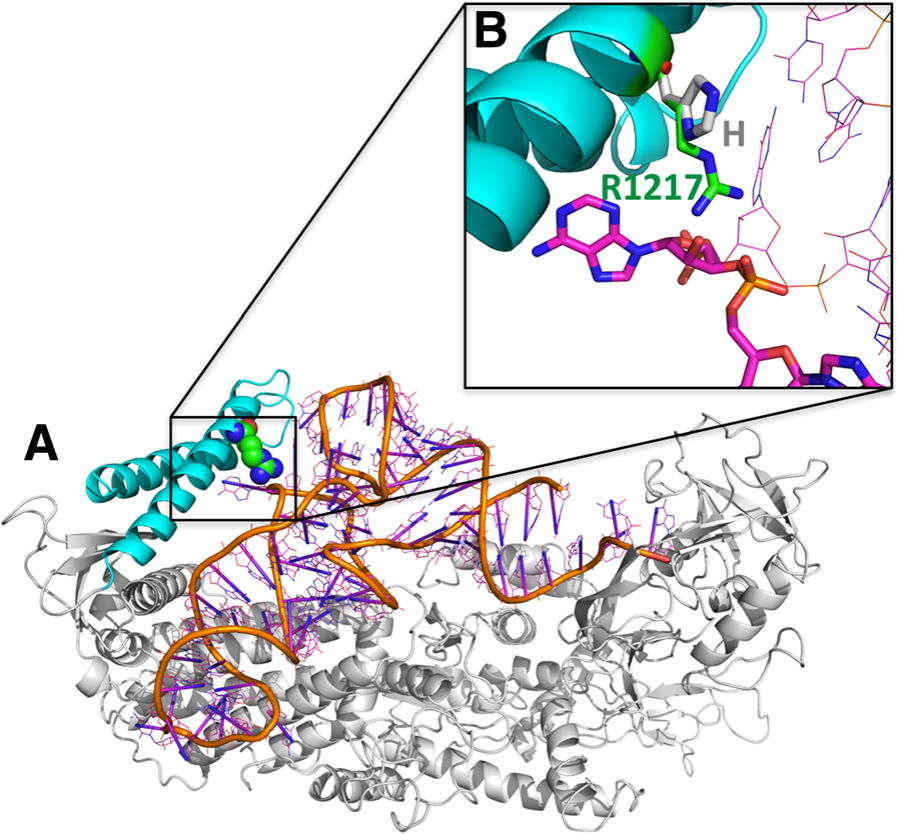
Computational structural analysis of mutant: Homology model of the VARS2 tRNA synthetase domain, based on the PDB id 1gax (36% sequence id, RaptorX *p* value 1.12e-19). **a** The C-terminal coiled-coild domain is shown in cyan. R1217 is highlighted with carbon atoms shown in green. tRNA is shown with orange backbone. **b** Inlay: Zoom of the indicated region. H1217 is shown with gray carbon atoms. The tRNA D loop is shown with carbon atoms in magenta

VARS2 belongs to the class-I aminoacyl-tRNA synthetase family. The aminoacyl-tRNA synthetase (also known as aminoacyl-tRNA ligase) catalyzes the attachment of an amino acid to its cognate transfer RNA molecule in a highly specific two-step reaction. Class I aminoacyl-tRNA synthetases contain a characteristic Rossman fold catalytic domain and are mostly monomeric. tRNA binding involves an alpha-helical structure that is conserved between class I and class II synthetases. R1217 is located in the C-terminal coiled-coil domain of the tRNA synthetase domain of VARS2 (Fig. 4a). R1217 directly binds to the phosphate backbone of the tRNA D- loop. R1217 also forms a hydrogen bond with the ribose moiety of an adenosine that is flipped out and points toward the coiled-coil domain (Fig. 4b). R1217 has therefore an important function in the specific recognition of tRNA. The mutation of R1217 into a histidine abolishes this function. Hence, the R1217H mutant is expected to have compromised abilities to recognize the correct tRNA. This is consistent with the prediction of pathogenicity of this variant by models including Polyphen, SIFT, and CADD.

### Discussion

Aminoacyl-tRNA synthetase enzymes (aaRSs) are involved in translation at the second step in creating a protein from a gene’s instructions. The mRNA interacts with the ribosomal complex which reads the sequence of mRNA bases. Each sequence codes for one particular amino acid, and tRNA assembles the protein, one amino acid at a time. Subsequently, aaRSs help attach the amino acid to the tRNA [14].

A typical animal cell contains 37 cytoplasmic and mitochondrial synthetase genes, all of which are encoded on nuclear chromosomes. Mitochondrial synthetases are given the same name as cytosolic synthetases, appended with the numeral 2 (e.g., HARS and HARS2) [15]. Mitochondrial and cytoplasmic aaRSs are encoded by distinct nuclear genes, with the exception of GARS and KARS, which are present in both cellular compartments [16]. Mt-tRNA genes are located in three transcription units: the short H-strand unit (with the rRNA region and two tRNA genes) is transcribed twice as fast as the L-strand that contains eight tRNA genes and more frequently the entire H-strand unit that produces 14 tRNAs [17]. Currently, pathological mutations that cause human diseases have been identified in many genes that encode cytoplasmic and mitochondrial tRNA-synthetases. The first cytoplasmic aaRSs mutation associated with a human disease, Charcot-Marie-Tooth, was discovered in glycyl-tRNA synthetase (GARS) [18]. Typically, defects in each mtARS have been identified in one tissue-specific disease (most commonly affecting the brain) or in one syndrome [19].

*VARS2* encodes mitochondrial valyl-tRNA (Val-tRNA) synthetase, which is involved in mitochondrial translation [16]. The deduced 993-amino acid protein has an N-terminal mitochondrial targeting signal with a predicted cleavage site after residue 29. VARS2 has characteristics of a class I mitochondrial aminoacyl-tRNA synthetase, including a classical Rossmann fold [20].

A homozygous mutation of *VARS2* was reported in a sporadic case with microcephaly and epilepsy [16]. It was associated with isolated deficiency of the mitochondrial respiratory chain (MRC) complex I. The disorder was secondary to a homozygous c.1100C>T transition in *VARS2*, resulting in a threonine 367 to isoleucine (T367I) substitution at a highly conserved residue. The affected patient was an 8-year-old with psychomotor delay, facial dysmorphisms, and microcephaly. At 4 years of age, there was onset of partial seizures. His brain MRI displayed hyperintense lesions in the periventricular regions, the insula, and the right fronto-temporal cortex. Brain proton magnetic resonance spectroscopy revealed a lactate peak in the frontal white matter. Muscle computerized tomography showed bilateral quadriceps hypotrophy, but no evident histological alteration was observed in a muscle biopsy [16].

In a British boy with combined oxidative phosphorylation deficiency-20, a compound heterozygous mutation of *VARS2*: a c.1135G>A transition, resulting in an alanine 379 to threonine (A379T) substitution, and a c.1877C-A transversion, resulting in an alanine 626 to aspartic acid substitution (A626D). He presented with progressive external ophthalmoplegia, ataxia, and combined oxidative phosphorylation deficiencies [21].

Our family share with the reported cases, the findings of microcephaly, epilepsy, and the progressive cerebral encephalopathy; however, several dysmorphic features were noticeable in clinical assessment of the affected members from our family. The hypogonadism, vitamin D resistant osteomalacia, and the growth hormone deficiency were significant and novel associations with the *VARS2* mutation in the family from this study (Table 1).

**Table 1:** Genetic and phenotypic heterogeneity

Clinical features	Diodato et al.	Taylor et al.	This study
	NM_001167734.1: c.1100C>T, p.Thr367Ile (Hom)	NM_001167734.1: c.1135G>A,p.Ala379Thr (Het) c.1877C>A, p.Ala626Asp (Het)	NM_006295: c.A3253G; p.S1085G
Mental retardation	X		X
Facial dysmorphisms	X		X
Ataxia		X	X
Combined oxidative phosphorylation deficiencies		X	
Microcephaly	X		X
Progressive external ophthalmoplegia		X	
Epilepsy	X		X
Short stature			X
Hyperintense lesions in the periventricular regions, the insula, and the right frontotemporal cortex	X		
Evidence of severe growth hormone deficiency			X
Hypogonadism			X
Severe osteomalacia			X

X : affected

In our *VARS2* family, there were no apparent deficiencies in mitochondrial electron transport chain enzymes. However, citrate synthase activity was increased, suggesting increased mitochondrial proliferation. We identified a homozygous change *VARS2:*NM_006295:c.3650G>A:R1217H, segregating with disease in our family. This mutation is in the anticodon-binding domain of tRNA synthetase, while the reported isolated *VARS2* homozygous mutation [16] is located in the catalytic core domain of valyl-tRNA synthetase.

The lack of clear biochemical phenotypes of OXPHOS or mitochondrial protein synthesis defects in skin fibroblasts and myoblasts from most of mutant aaRSs patients prevented the use of cultured cells for functional studies. In a few RARS2 patients, fibroblasts showed OXPHOS defects, with RC enzymes variably affected; YARS2 patients presented deficient mitochondrial protein synthesis in myotubes but not in cultured fibroblasts [22].

### Conclusions

In summary, we describe a novel autosomal recessive syndromic disorder associated with a mutation in *VARS2* (mitochondrial valyl tRNA synthetase). Clinical features included severe mental retardation, ataxia, speech impairment, epilepsy, short stature, microcephaly, dysmorphia, an easily excitable personality, and excessive chewing mouth behaviors. In addition, there was clinical and chemical evidence of severe growth hormone deficiency, hypogonadism, and severe osteomalacia. Our findings further expand the phenotypic spectrum of Aminoacyl-tRNA synthetase enzymes.

## References

[1] Al-Mujadi H, AR AR, Katzarov MG, Dehrab NA, Batra YK, Al-Qattan AR (2006). Preemptive gabapentin reduces postoperative pain and opioid demand following thyroid surgery. Can J Anaesth.

[2] Fernell E (1998). Aetiological factors and prevalence of severe mental retardation in children in a Swedish municipality: the possible role of consanguinity. Dev Med Child Neurol.

[3] Ropers HH (2010). Genetics of early onset cognitive impairment. Annu Rev Genomics Hum Genet.

[4] de Ligt J, Willemsen MH, van Bon BW, Kleefstra T, Yntema HG, Kroes T, Vulto-van Silfhout AT, Koolen DA, de Vries P, Gilissen C (2012). Diagnostic exome sequencing in persons with severe intellectual disability. N Engl J Med.

[5] Fryns JP, de Ravel TJ (2002). London Dysmorphology Database, London Neurogenetics Database and Dysmorphology Photo Library on CD-ROM [Version 3] 2001R. M. Winter, M. Baraitser, Oxford University Press, ISBN 019851-780, pound sterling 1595. Hum Genet.

[6] Ramsden SC, Clayton-Smith J, Birch R, Buiting K (2010). Practice guidelines for the molecular analysis of Prader-Willi and Angelman syndromes. BMC Med Genet.

[7] Tan WH, Bird LM, Thibert RL, Williams CA (2014). If not Angelman, what is it? A review of Angelman-like syndromes. Am J Med Genet A.

[8] Arnold K, Bordoli L, Kopp J, Schwede T (2006). The SWISS-MODEL workspace: a web-based environment for protein structure homology modelling. Bioinformatics.

[9] Kallberg M, Margaryan G, Wang S, Ma J, Xu J (2014). RaptorX server: a resource for template-based protein structure modeling. Methods Mol Biol.

[10] Yang J, Yan R, Roy A, Xu D, Poisson J, Zhang Y (2015). The I-TASSER Suite: protein structure and function prediction. Nat Methods.

[11] Kall L, Krogh A, Sonnhammer EL (2004). A combined transmembrane topology and signal peptide prediction method. J Mol Biol.

[12] UniProt C (2015). UniProt: a hub for protein information. Nucleic Acids Res.

[13] Hunter S, Jones P, Mitchell A, Apweiler R, Attwood TK, Bateman A, Bernard T, Binns D, Bork P, Burge S (2012). InterPro in 2011: new developments in the family and domain prediction database. Nucleic Acids Res.

[14] Francklyn C, Musier-Forsyth K, Martinis SA (1997). Aminoacyl-tRNA synthetases in biology and disease: new evidence for structural and functional diversity in an ancient family of enzymes. RNA.

[15] Abbott JA, Francklyn CS, Robey-Bond SM (2014). Transfer RNA and human disease. Front Genet.

[16] Diodato D, Melchionda L, Haack TB, Dallabona C, Baruffini E, Donnini C, Granata T, Ragona F, Balestri P, Margollicci M (2014). VARS2 and TARS2 mutations in patients with mitochondrial encephalomyopathies. Hum Mutat.

[17] King MP, Attardi G (1993). Post-transcriptional regulation of the steady-state levels of mitochondrial tRNAs in HeLa cells. J Biol Chem.

[18] Antonellis A, Ellsworth RE, Sambuughin N, Puls I, Abel A, Lee-Lin SQ, Jordanova A, Kremensky I, Christodoulou K, Middleton LT (2003). Glycyl tRNA synthetase mutations in Charcot-Marie-Tooth disease type 2D and distal spinal muscular atrophy type V. Am J Hum Genet.

[19] Euro L, Konovalova S, Asin-Cayuela J, Tulinius M, Griffin H, Horvath R, Taylor RW, Chinnery PF, Schara U, Thorburn DR (2015). Structural modeling of tissue-specific mitochondrial alanyl-tRNA synthetase (AARS2) defects predicts differential effects on aminoacylation. Front Genet.

[20] Bonnefond L, Fender A, Rudinger-Thirion J, Giege R, Florentz C, Sissler M (2005). Toward the full set of human mitochondrial aminoacyl-tRNA synthetases: characterization of AspRS and TyrRS. Biochemistry.

[21] Taylor RW, Pyle A, Griffin H, Blakely EL, Duff J, He L, Smertenko T, Alston CL, Neeve VC, Best A (2014). Use of whole-exome sequencing to determine the genetic basis of multiple mitochondrial respiratory chain complex deficiencies. JAMA.

[22] Diodato D, Ghezzi D, Tiranti V (2014). The mitochondrial aminoacyl tRNA synthetases: genes and syndromes. Int J Cell Biol.

