# Correction to: Recessive VARS2 mutation underlies a novel syndrome with epilepsy, mental retardation, short stature, growth hormone deficiency, and hypogonadism

**DOI:** 10.1186/s40246-017-0130-6

**Published:** 2017-12-08

**Authors:** Abdulaziz Alsemari, Banan Al-Younes, Ewa Goljan, Dyala Jaroudi, Faisal BinHumaid, Brian F. Meyer, Stefan T. Arold, Dorota Monies

**Affiliations:** 1 Department of Neurosciences, King Faisal Specialist Hospital and Research Centre, Riyadh, Saudi Arabia; 2 Department of Genetics, King Faisal Specialist Hospital and Research Centre, MBC 03, PO Box 3354, Riyadh, 11211 Saudi Arabia; 3 Saudi Human Genome Project, King Abdulaziz City for Science and Technology, Riyadh, Saudi Arabia; 4 King Abdullah University of Science and Technology (KAUST), Computational Bioscience Research Center (CBRC), Division of Biological and Environmental Sciences and Engineering (BESE), Thuwal, 23955-6900 Saudi Arabia

## Correction

After publication of the article [1], it has been brought to our attention that there is a nomenclature issue with this article. At the time of acceptance, the VARS2 mutation was considered equivalent to the VARS mutation. However, this has changed so that VARS2 now only refers to shorter mitochondrial sequence of valyl-tRNA synthesase containing 1093 amino acids. Therefore, in the context of this article, every usage of *“VARS2”* should be replaced with *“VARS”* when referring to the causative variant.
